# Characterizing a strong pan-muscular promoter (P*mlc-1*) as a fluorescent co-injection marker to select for single-copy insertions

**DOI:** 10.17912/micropub.biology.000302

**Published:** 2020-09-03

**Authors:** Sonia El Mouridi, Yuli Peng, Christian Frøkjær-Jensen

**Affiliations:** 1 King Abdullah University of Science and Technology (KAUST), Biological and Environmental Science and Engineering Division (BESE), KAUST Environmental Epigenetics Program (KEEP), Thuwal, 23955-6900, Saudi Arabia

**Figure 1. Using a pan-muscular promoter for single-copy transgene selection:**
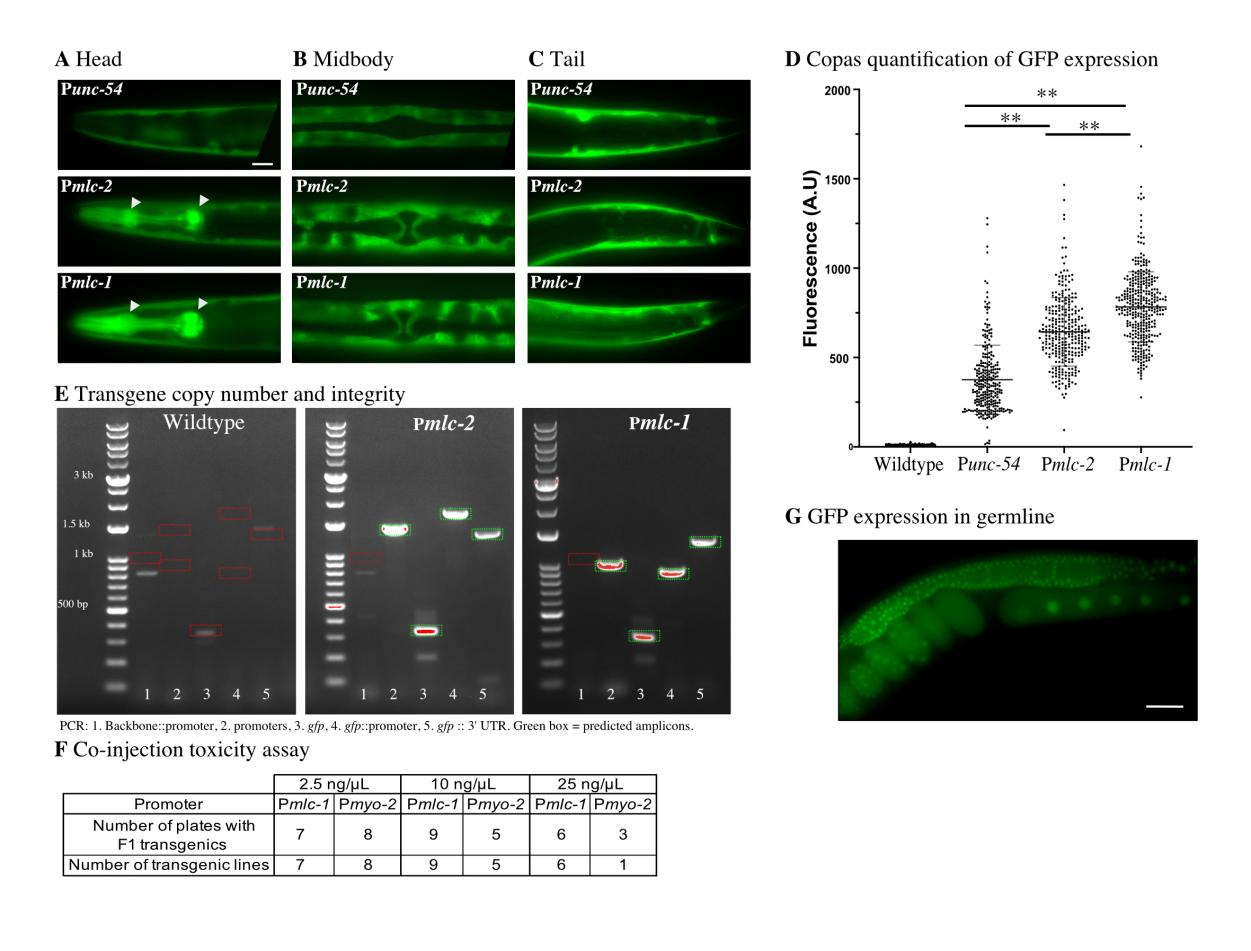
**A-C.** Characterization of the fluorescence expression pattern from MosSCI insertions of transgenes with P*unc-54::gfp*, P*mlc-2::gfp*, or P*mlc-1::gfp*. We acquired images at 20x magnification from immobilized young adult animals. Scale bar = 20 microns. **A.** Expression in the head region. All transgenes were expressed in head muscles, but only P*mlc-1* and P*mlc-2* were expressed in pharyngeal muscles (indicated by white arrowheads). **B.** In the vulval region, all promoters expressed GFP in body wall muscles. Only P*mlc-1* and P*mlc-2* were expressed in vulval muscles. **C.** In the tail region, all promoters expressed GFP in body wall muscles, stomato-intestinal and anal depressor muscles, **D.** Quantification of GFP expression in young adult animals by flow cytometry (Copas) in MosSCI strains expressing P*unc-54::gfp*, P*mlc-2::gfp* and P*mlc-1::gfp*. One-way ANOVA with Sidak’s multiple comparison test (N2 (N= 293), P*unc-54* (N=277), P*mlc-1* (N = 301), P*mlc-2* (N=346)). **E.** Screen for transgene copy number. PCR primers were designed to amplify (1) the vector backbone which is commonly duplicated for dual inserts (950 bp), (2) the transgene promoters (1.5 kb for P*mlc-2* and 1.0 kb for P*mlc-1*), (3) the *gfp* fluorophore (344 bp), (4) the *gfp* and promoter junctions (1.8 kb for P*mlc-2* and 945 bp for P*mlc-1*), and (5) the junction between *gfp* and 3′ UTR (1.4 kb). Green boxes indicate expected bands, and red boxes indicate controls with no expected PCR amplification. The band in lane 3 from wildtype DNA is likely due to minor contamination. No dual or “complex” inserts were detected. **F.** Transgenes were tested for toxicity by injection at increasing concentrations in ten animals (2.5, 10, or 25 ng/uL) using P*mlc-1* or P*myo-2* with an mCherry fluorescent marker. **G.** Picture at 40x magnification of a P*smu-1*:*gfp* MosSCI insertion used to test P*mlc-1* as a single co-injection marker. As expected, GFP was expressed strongly in the germline and less in somatic cells (not shown). Scale bar = 20 microns.

## Description

Fluorescent markers are useful for identifying transgenic *C. elegans* after injection. In some cases, fluorophores are used to identify transgenics and to propagate animals with extra-chromosomal or integrated arrays (*e.g.*, *sur-5*::*gfp*) (Gu *et al.* 1998). In other cases, fluorescent markers are used as visual markers to identify and later select against array animals. Such negative selection is used generate single-copy transgene insertions by plasmid injection, *e.g.*, Mos1-mediated single-copy insertion (MosSCI)(Frøkjær-Jensen *et al.* 2008) or CRISPR/Cas9 (Dickinson *et al.* 2013). These methods generate targeted double-strand breaks, and transgenes are inserted by homologous recombination into specific locations. Selection schemes that rely on positive (*e.g.*, *cbr-unc-119*, *NeoR*, *HygroR*)(Maduro and Pilgrim 1995; Giordano-Santini *et al.* 2010; Radman *et al.* 2013) and negative (*e.g.*, fluorophores or the *peel-1* toxin) (Frøkjær-Jensen *et al.* 2012) selection markers are frequently used to identify single-copy insertions. Negative *peel-1* selection is under control of a heat-shock promoter (P*hsp-16.41*); after heat-shock, animals with arrays or dual insertions with the *peel-1* transgene rapidly die (Seidel *et al.* 2011). *peel-1* is convenient, but this selection has two significant drawbacks: the transgene is toxic in the absence of heat shock, resulting in fewer F1 progeny (Frøkjær-Jensen *et al.* 2012), and the induced lethality is often not fully penetrant resulting in false positives. Regardless of whether the *peel-1* selection is used, we have advocated for the inclusion of all three red fluorescent markers expressed in neurons (P*rab-3*), body-wall muscle (P*myo-3*), and pharynx (P*myo-2*) because a single or even two markers were inefficient at avoiding false positives. These three selection markers are widely used and have been requested more than 300 times each from Addgene. However, adding three fluorescent markers to every injection mix is increasingly inconvenient as the mixes also contain plasmids encoding enzymes (*Mos1* transposase or Cas9), sgRNAs, repair templates, and sometimes additional markers. Pharyngeal expression of the P*myo-2* fluorophore is the easiest to identify on a fluorescence dissection microscope due to early embryonic expression and brightness, but the transgene is frequently toxic and is therefore not commonly injected at high concentrations. For these reasons, we sought to identify an improved co-injection marker. An ideal marker would be bright, non-toxic, expressed in a clearly identifiable tissue, and only require a single marker in the injection mix. Here we demonstrate that fluorescent transgenes containing the pan-muscular promoter from myosin light chain 1 (*mlc-1*) fulfill these criteria. The *mlc-1* and the related *mlc-2* promoter may also be generally useful to identify all (body wall, pharynx, vulval, stomato-intestinal, and anal depressor) muscles or for robust expression of transgenes (*e.g.*, optogenetic sensors).

We focused on promoters from genes expected to have muscle-specific expression and manually checked their relative expression levels based on RNA-seq from the ModENCODE project (Gerstein *et al.* 2010). We identified two putative muscle-specific myosin light chain genes, *mlc-1* and *mlc-2*, that are highly expressed from a single divergent locus. RNA expression levels based on Fragments Per Kilobase Million reads (FPKM) are comparatively high for these two genes in young adults, with 500 FPKM (*mlc-1*) and 1159 FPKM (*mlc-2*) compared to 230 FPKM (*unc-54*), 14 FPKM (*myo-2*), and 29 FPKM (*myo-3*). We selected *unc-54*, *mlc-1*, and *mlc-2* as candidates and amplified 1.9 kb, 1.3 kb, and 1.2 kb promoters, respectively. Due to an early splice site (immediately after the start codon), P*mlc-1* also included the first six amino acids of *mlc-1*. To quantify the relative expression level of the three promoters, we generated otherwise identical transgenes with a codon-optimized *gfp* under each promoter’s control and made single-copy insertions at the *ttTi5605* site using MosSCI. All promoters drove GFP expression in the body wall, stomato-intestinal, and anal depressor muscles (**Figure 1A-C**). However, only P*unc-54* was not expressed in pharyngeal (**Figure 1A**) and vulval muscles (**Figure 1B**) in agreement with previous promoter analysis (Okkema *et al.* 1993). Using a Copas large-particle flow cytometer, we quantified GFP expression in young adults from a mixed population of the MosSCI strains and found that P*mlc-1*:*gfp* resulted in the highest expression, with approximately two-fold more expression than P*unc-54* (**Figure 1D**). The fluorescence signal matched our subjective visual impression from transgenic animals carrying extra-chromosomal arrays. We verified that the P*mlc-1* and P*mlc-2* strains harbored only single transgene insertions (**Figure 1E**), excluding the possibility that transgene copy-number caused the observed difference in fluorescence.

Co-injection markers are incorporated into extra-chromosomal arrays in proportion to their concentration in the injection mix (Mello *et al.* 1991). Higher concentrations thus make it less likely to generate array animals lacking the fluorescent marker, which is essential to avoid false positives (*i.e.*, rescued animals with no single-copy transgene inserted). Therefore, we quantified the toxicity of P*mlc-1:*:*mCherry* and P*myo-2:*:*mCherry* transgenes when used as co-injection markers at increasing concentrations (2.5 ng/ul, 10 ng/ul, and 25 ng/ul). We quantified the number of injected P0 animals that gave F1 transgenic animals and stable F2 transgenic lines (**Figure 1F**). We did not observe apparent toxicity from either promoter at low concentrations (2.5 ng/ul and 10 ng/ul). At high concentration (25 ng/ul), we observed a reduction in plates with rescued F1s and fewer stable lines for P*myo-2*, suggesting some toxicity. In contrast, even at high concentrations, P*mlc-1* did not show any apparent toxicity.

Our goal was to use a single, bright co-injection marker to generate MosSCI insertions, and the lack of toxicity allowed us to test P*mlc-1* at high concentration (25 ng/ul). We inserted a germline-expressed P*smu-1*::*gfp* transgene (Spike *et al.* 2001) by MosSCI using *cbr-unc-119* as the positive selection and a P*mlc-1::tagRFP-T* transgene as the visual negative selection marker (in addition to P*hsp-16.41*:*peel-1*). We injected ten animals and recovered six plates with extra-chromosomal arrays in the F2 or F3 generation. We heat-shocked these plates and identified four plates with rescued animals (*i.e.*, animals with normal movement) but with no visible red fluorescence on a dissection fluorescence microscope. To rule out false positives, we picked a single worm from each plate and checked for stable germline expression from a transgene insert in the next generation (**Figure 1G**). All four independent lines expressed GFP in the germline and we observed no *tagRFP* expression from the fluorescent co-injection marker at high magnification. These results indicate that we could use a single fluorescence marker to select against false positives. Homologous repair relies on unique sequences in the extra-chromosomal array. Therefore, we have generated a set of green (*gfp* and *mNeonGreen*) and red (*mCherry* and *TagRFP-T*) P*mlc-1* fluorescent markers with cytoplasmic and nuclear expression to avoid cross-talk with the repair machinery.

Here, we describe two promoters from *mlc-1* and *mlc-2* that can drive bright fluorescent expression in all muscles. We have used *mlc-1* to generate co-injection markers that are non-toxic at high concentrations. The use of a single fluorescent co-injection marker solves a minor inconvenience when generating MosSCI injection mixes and would, presumably, also be useful for other methods that rely on similar selection schemes. More generally, *mlc-1* and *mlc-2* promoters should be generally useful when a bright pan-muscle expression is desired, *e.g.*, for transgene rescue, for expressing optogenetic effectors (e.g., Kerr *et al.* 2000), or for isolating cells or nuclei from all muscles (e.g., Serizay *et al.* 2020). P*mlc-2* is expressed at slightly lower levels but may be preferable in the cases where the first six amino acids of *mlc-1* might perturb experiments. To facilitate the use of these reagents, we have deposited fluorescent transgenes and Gateway-compatible promoter vectors at Addgene for distribution.

## Methods

Reagents

Co-injection markers available at Addgene

pSEM228 – *Pmlc-1::mNeonGreen(2xNLS)::cbr-tbb-2 UTR* (Addgene #159895)

pSEM229 – *Pmlc-1::mNeonGreen::cbr-tbb-2 UTR* (Addgene #159896)

pSEM230 – *Pmlc-1::GFP(2xNLS):: cbr-tbb-2 UTR* (Addgene # 159794)

pSEM231 – *Pmlc-1::GFP::cbr-tbb-2 UTR* (Addgene #159897)

pSEM232 – *Pmlc-1::TagRFP-T(2xNLS)::cbr-tbb-2 UTR* (Addgene #159898)

pSEM233 – *Pmlc-1::TagRFP-T::cbr-tbb-2 UTR* (Addgene #159899)

pSEM234 – *Pmlc-1::mCherry(2xNLS)::cbr-tbb-2 UTR* (Addgene # 159795)

pSEM235 – *Pmlc-1::mCherry::cbr-tbb-2 UTR* (Addgene #159900)

pCFJ2113 – [4-1] – *Promoter – Pmlc-1 (w ATG)* (Addgene #159888)

pSEM223 – [4-1] – *Promoter – Pmlc-2 (no start)* (Addgene #159889)

Annotated plasmid sequences are available at www.wormbuilder.org and https://www.addgene.org/Christian_Froekjaer-Jensen/

Molecular biology

We generated all co-injection markers by three-fragment multisite Gateway reactions (Invitrogen Cat #12538200). The pENTR[4-1] vectors were made by amplifying the promoters and assembled by BP Gateway reaction (Invitrogen Cat #11789013) into a custom pDONR4-1 vector (pCFJ1509). All PCRs were done using high fidelity Phusion polymerase (F530S) and validated by Sanger sequencing.

Promoter quantification

Transgenes with P*mlc-1*, P*mlc-2*, or P*unc-54* fused to a codon-optimized *gfp* were inserted by MosSCI into the EG4322 *(ttTi5605 ; unc-119(ed3))* strain following standard protocols. The injection mixes were composed of 25 ng/ul repair template, 20 ng/ul pCFJ104 (*Pmyo-3::mCherry*), 20 ng/ul pGH8 (*Prab-3::mCherry*), 5 ng/ul pCFJ90 (*Pmyo-2::mCherry*), 20 ng/ul pMA122 (*peel-1::HS*), 20 ng/ul pNP403 (histamine selection), 20 ng/ul pCFJ1532 (P*smu-1*:*Mos1* transposase). After injection, we singled each worm onto a standard NGM plate seeded with HB101 and placed animals at 25º Celsius. Starved plates with *unc-119* rescued animals were placed for one hour at 37º Celsius (heat-shock) and “chunked” to a new NGM plate. Twenty-four hours later, we picked a single worm with *unc-119* rescue and without visible fluorescence from co-injection markers to generate a stable line. Once a line was generated, we placed worms onto standard NGM plates seeded with OP50 and grew at 25º Celsius.

Plasmids used to generate MosSCI insertion strains

pSEM218 – *Punc-54::gfp::cbr-tbb-2 UTR*

pSEM219 – *Pmlc-2::gfp::cbr-tbb-2 UTR*

pSEM220 – *Pmlc-1::gfp::cbr-tbb-2 UTR*

MosSCI strains

CFJ62: *kstSi26*[P*unc-54* | *gfp* | *cbr-tbb-2* 3′ UTR] II; *unc-119(ed3)* III

CFJ63: *kstSi27*[P*mlc-2* | *gfp* | *cbr-tbb-2* 3′ UTR] II; *unc-119(ed3)* III

CFJ64: *kstSi28*[P*mlc-1* | *gfp* | *cbr-tbb-2* 3′ UTR] II; *unc-119(ed3)* III

To test if a single co-injection marker was sufficient to select for MosSCI insertions, we used the same strategy but with a germline-expressed transgene and a modified injection mix. The mix consisted of 25 ng/ul pCFJ1805 (P*smu-1*::gfp) for germline expression, 25 ng/ul pSEM233 (*Pmlc-1::TagRFP-T*), 20 ng/ul pMA122 (*peel-1 ::HS*), 20 ng/ul pCFJ1532 (P*smu-1*:*Mos1* transposase), GeneRuler 1kb plus DNA ladder (ThermoFisher SM1331) for a final concentration of 100 ng/ul.

Fluorescence quantification

We placed six worms onto NGM plate seeded with OP50 (four plates per strain) and placed animals at 25º Celsius. After five days, worms were washed off growth plates with M9 and washed twice to remove bacteria. We quantified fluorescence using a Copas flow cytometer (Union Biometrica). A mixed population was analyzed, but we only quantified the fluorescence of young adult worms (based on “time of flight,” *i.e.*, animal length).

Transgene toxicity analysis

Transgenic worms were generated by injection into N2 (wild type) worms. The injection mixes were composed of P*mlc-1* or P*myo-2* fused to mCherry at different concentrations (2.5 ng/ul ; 10 ng/ul ; 25 ng/ul) and GeneRuler 1kb plus DNA ladder (ThermoFisher SM1331) for a final concentration of 100 ng/ul. Ten worms were injected per condition, singled onto NGM plates seeded with OP50, and placed at 25º Celsius. 48 hours after, we determined the number of plates with transgenic F1s and several days later determined if the plates had stable arrays lines in the F2 or F3 generation.

Plasmids used for toxicity assays

pSEM235 – P*mlc-1::mCherry*

pCFJ90 – P*myo-2::mCherry*

Microscopy

We imaged animals on a Leica DM2500 microscope equipped with a Leica DFC7000 GT camera at 20X. Worms were immobilized using an M9 solution containing 50 mM of sodium-azide and mounted on 2% agarose pads. We analyzed the fluorescence signal using ImageJ and Affinity designer software.
